# A Closer Examination of Sore Throats and Their Hidden Dangers: A Case Report of Lemierre’s Syndrome

**DOI:** 10.7759/cureus.111558

**Published:** 2026-06-26

**Authors:** Sriram Nallamothu, Darius Jaimungal, Joseph Zackerali, Kavitha Duggineni, Christopher Chang

**Affiliations:** 1 Department of Internal Medicine, South-West Regional Health Authority, Point Fortin, TTO; 2 Department of Radiology, South-West Regional Health Authority, Point Fortin, TTO; 3 Department of Accident and Emergency, South-West Regional Health Authority, Point Fortin, TTO; 4 Department of Primary Health Care, South-West Regional Health Authority, San Fernando, TTO

**Keywords:** cervical pain, diagnostic delay, internal jugular vein thrombophlebitis, lemierre’s syndrome (ls), oropharyngeal infection, septic emboli

## Abstract

Lemierre’s syndrome is a rare but potentially fatal complication of acute oropharyngeal infection characterized by internal jugular vein septic thrombophlebitis and metastatic septic embolization. We report a case of a 40-year-old man who initially presented with symptoms consistent with a viral upper respiratory tract infection and mild sore throat that progressed over several days to fever, myalgia, and right-sided facial and cervical pain. Within 48 hours, he developed internal jugular vein thrombosis and septic emboli on ultrasound and confirmed on contrast-enhanced computed tomography, supporting a diagnosis of Lemierre’s syndrome.

The patient was managed with intravenous antibiotics and anticoagulation, resulting in complete clinical recovery. This case highlights the diagnostic challenge posed by early nonspecific symptoms and emphasizes the need for a high clinical index of suspicion, swift recognition, early imaging, and early initiation of treatment to achieve favorable outcomes, particularly in settings where endemic infections may obscure the diagnosis of Lemierre’s syndrome.

## Introduction

Lemierre’s syndrome is a rare but potentially life-threatening complication of acute oropharyngeal infection. It is characterized by septic thrombophlebitis of the internal jugular vein following the spread of infection from the oropharynx to the parapharyngeal space, often resulting in septic emboli [1]. The condition was first described by Lemierre, a French bacteriologist, in a case series of 20 patients who developed septicemia linked to anaerobic microorganisms [2,3].

Since then, Lemierre’s syndrome has been clinically defined by the presence of a recent oropharyngeal infection, along with clinical or radiological evidence of internal jugular vein thrombosis with a causative anaerobic pathogen, commonly *Fusobacterium necrophorum*.

*F. necrophorum* is a nonmotile, anaerobic, non-spore-forming, gram-negative bacterium that can be a part of the normal oropharyngeal flora. Although a rare condition, with an estimated global incidence of approximately one per 1,000,000, Lemierre’s syndrome predominantly affects healthy adolescents and young adults with a median age range from 19 to 22 years, with approximately 90% of cases arising in individuals aged between 10 and 35 years. A male predominance is consistently reported, with a male-to-female ratio of approximately 2:1 [4].

We present a case of a young adult male who initially presented with a seemingly uncomplicated sore throat that subsequently progressed to Lemierre’s syndrome, highlighting the diagnostic challenges and importance of early recognition despite nonspecific features, but ultimately resulting in a favorable outcome.

## Case presentation

A 40-year-old male air conditioning technician presented to the Accident and Emergency Department with a five-day history of low-grade fever, generalized myalgia, fatigue, and sore throat. He had initially presented to a primary care facility, where a diagnosis of upper respiratory tract infection was made, and was prescribed paracetamol and oral antibiotics with subsequent follow-up. Two days following his initial evaluation, his condition deteriorated, and he developed new symptoms, including diarrhea, chills, and localized right-sided facial and cervical pain, and was subsequently admitted for further inpatient evaluation and management.

His past medical history was unremarkable. He denied any recent tobacco or alcohol use and reported no use of herbal medication, although he admitted to cannabis use one week prior to presentation. There was no history of recent sickness, prior hospitalizations, or surgical procedures. Immunization records were up to date.

Initial laboratory investigations revealed leukocytosis with marked thrombocytopenia. C-reactive protein was significantly elevated. Renal function test indicated acute kidney injury while electrolytes remained within normal limits. Liver function tests were mildly deranged, with elevated aspartate aminotransferase and alanine aminotransferase (Table 1). The electrocardiogram demonstrated sinus tachycardia, and the chest radiograph was unremarkable.

**Table 1:** Laboratory parameters on initial presentation BUN: blood urea nitrogen, AST: aspartate aminotransferase, ALT: alanine aminotransferase, ALP: alkaline phosphatase, GGT: gamma-glutamyl transferase, CRPhs: high sensitivity C-reactive protein, PT: prothrombin time, aPTT: activated partial thromboplastin time, INR: international normalized ratio

Blood parameters	Patient values	Reference range
Hemoglobin	13.9	11.7-15.5 g/dL
White blood cells	20.8	4.1-11.2 × 10^3 ^uL
Platelets	65	159-388 × 10^3 ^uL
Sodium	138	135.0-145.0 mmol/L
Potassium	3.5	3.5-5.1 mmol/L
Chloride	102.2	97.0-110.0 mmol/L
Creatinine	2.5	0.7-1.2 mg/dL
BUN	28	6-23 mg/dL
AST	44.3	5.0-40.0 IU/L
ALT	90.8	5.0-41.0 IU/L
ALP	79	40-129 U/L
GGT	37	6.00-61.00 U/L
Total bilirubin	0.9	0.00-1.20 mg/dL
Direct bilirubin	0.41	0.00-0.40 mg/dL
Indirect bilirubin	0.49	0.00-1.00 mg/dL
CRPhs	36.9	0.100-0.500 mg/dL
PT	17.7	11.0-18.0 seconds
aPTT	35.6	27.0-35.0 seconds
INR	1.2	0.8-1.2

On the second day of admission, the patient reported worsening sore throat. Examination revealed tender anterior and posterior cervical lymphadenopathy without tonsillar exudate or pharyngeal inflammation. An ultrasonogram of the right lateral neck was then performed, which demonstrated multiple enlarged reactive lymph nodes, the largest measuring 1.8 cm × 0.5 cm. There was thickening of the right internal jugular vein wall with low-level intraluminal echoes and absent color Doppler flow, consistent with thrombus formation (Figure 1).

**Figure 1:**
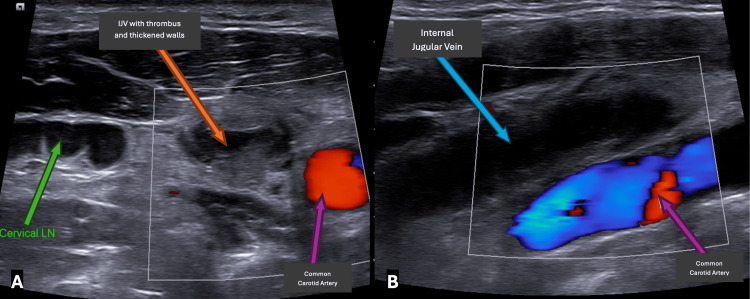
(A) Axial ultrasound of the right neck demonstrating enlarged cervical lymph nodes (green arrow), thickened IJV walls with thrombus material within the lumen (orange arrow), and the common carotid artery (purple arrow). (B) Doppler ultrasound demonstrating the absence of flow within the right internal jugular vein (blue arrow), with the common carotid artery indicated by a purple arrow IJV: internal jugular vein

In view of these findings, a differential diagnosis of Lemierre’s syndrome was strongly suspected, and further imaging was performed. Computed tomography (CT) imaging of the brain, neck, and chest with contrast, along with an arteriogram and venogram, was requested, which revealed a 5.1 cm (longitudinal size) × 1.1 cm (anteroposterior) × 0.6 cm (transverse (TS)) thrombus completely occluding the right internal jugular vein, extending from the C1 to the C4 level with extension into the proximal-to-mid right common facial vein. The walls of the internal jugular and common facial veins at the level of the thrombus were thickened and enhanced, where the internal jugular walls measure 0.3 cm (TS) and the common facial vein walls measure 0.2 cm (TS), consistent with an underlying thrombophlebitis (Figure 2).

**Figure 2:**
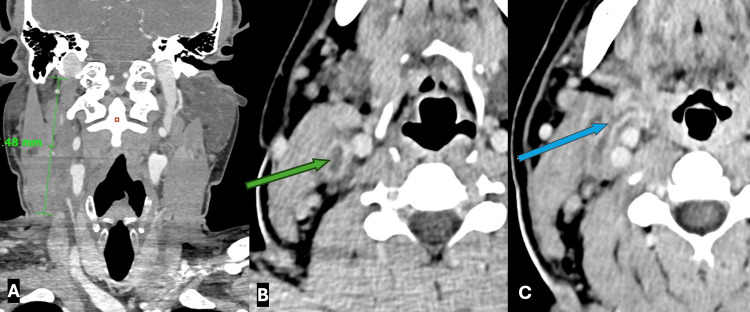
(A) Coronal CT imaging of the neck showing a large filling defect within the right internal jugular vein. Contrast opacification is seen superiorly within the jugular foramen and inferiorly within the distal aspect of the internal jugular vein, at the level where the internal jugular vein joins the subclavian vein. (B) Axial CT imaging demonstrating the right internal jugular vein with enhancing walls and a filling defect within (green arrow). (C) Axial CT imaging showing the right facial vein with enhancing walls and a filling defect within (blue arrow) CT: computed tomography

CT chest revealed multiple small opacifications measuring <2 cm (TS) throughout both lung fields with a predominantly subpleural distribution. Some of these opacifications showed central cavitation (Figure 3A). Additionally, subpleural micronodules were noted within both lung fields (Figure 3B). These findings were consistent with septic emboli.

**Figure 3:**
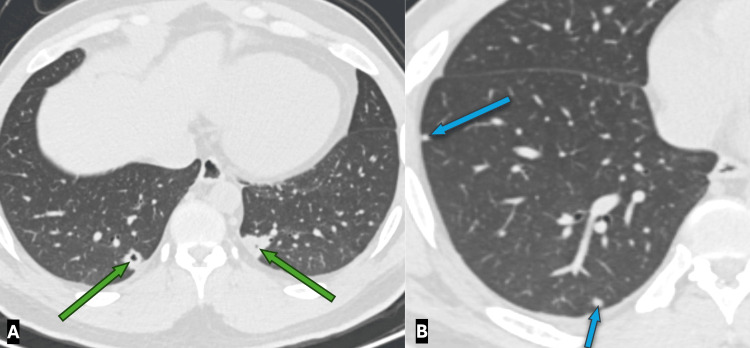
(A) Axial CT imaging of both lung fields showing bilateral peripherally distributed nodules with central cavitations abutting the pleura (green arrows). (B) Axial CT imaging of the right lung base showing subpleural micronodules (blue arrows) CT: computed tomography

Coagulation studies were within normal limits (Table 1). Testing for HIV, dengue, and leptospirosis was negative. Bedside echocardiography revealed no vegetations or thrombus. Urine microscopy, culture, and sensitivity showed no bacterial growth. Autoimmune serology, blood culture, and sensitivity were not available.

Treatment

Initial management included fluid resuscitation and intravenous ceftriaxone, which was continued for five days and resulted in gradual clinical improvement. The patient experienced persistent facial and cervical pain 48 hours after initiation of treatment, which was managed symptomatically with acetaminophen and tramadol. Anticoagulation with warfarin was commenced with regular international normalized ratio monitoring, and the patient was subsequently discharged on oral anticoagulation therapy.

Outcome and follow-up

On admission, the patient was noted to have an acute kidney injury with a creatinine rise to 2.5 mg/dL, which resolved during the course of treatment. The overall hospital stay was otherwise uneventful, and the patient achieved full clinical recovery by day 14. On discharge, the patient was prescribed rivaroxaban 15 mg twice daily for three weeks, followed by 20 mg once daily for three months, and was scheduled for follow-up at the medical outpatient clinic.

## Discussion

Given the severity of Lemierre's syndrome, clinicians should maintain a high index of suspicion when dealing with cases of acute throat infection, especially in situations where symptoms appear atypical or worsening. A preceding history of a sore throat, fever, neck pain, and other concerning signs should prompt early consideration of this diagnosis, as timely recognition and initiation of treatment are critical for favorable outcomes.

In our patient's case, he initially presented with myalgia, fever, and generalized weakness. However, by the second day of admission, he developed facial pain and cervical tenderness. In this clinical context, endemic infections within this region, such as leptospirosis and dengue, initially confounded the diagnosis and were considered, which were further supported by indicators such as thrombocytopenia, declining platelet count trends, and deranged liver transaminase levels.

Common clinical features of Lemierre's syndrome include a sore throat, often preceding other symptoms, in 82.5% of cases, while fever is almost universal, reported in 82.5%-100% of patients. Cervical involvement, including neck swelling and tenderness, occurs in around 52.2% of cases, and jaundice which has been described in approximately one-third (33%) of patients [5-8].

In this case, blood culture could not be obtained due to resource limitations. *F. necrophorum*, the most frequently identified pathogen, is an obligate anaerobic, Gram-negative bacillus that may require up to seven days to manifest growth in blood culture and can sometimes be confused with Bacteroides species [9-11]. Its role as a component of normal oral flora remains debated. Negative blood cultures do not exclude Lemierre’s syndrome, particularly following prior antibiotic exposure. In addition, microbiological confirmation is not essential for diagnosis, as culture negativity is well recognized in a substantial proportion of cases.

The management involved intravenous antimicrobial therapy, with appropriate anaerobic coverage, as outlined by the recommendations of Lu et al. [12] and Riordan [13] and concurred by various authors.

Although our patient showed marked improvement in renal function within 48 hours, inflammatory markers such as C-reactive protein continued to rise. On day 6, anticoagulation was commenced. The role of anticoagulation remains controversial and is primarily prescribed for patients who do not exhibit a positive response to initial antibiotic treatment or in cases where there is a progression of thrombosis, including retrograde cavernous sinus thrombosis or thrombophilia [14,15]. In this case, anticoagulation was initiated due to documented extension of the thrombus into the proximal and mid right common facial vein.

Our patient also developed pulmonary involvement with multiple septic emboli, a well-recognized and serious complication of Lemierre's syndrome occurring in up to 97% of cases and primarily attributed to hematogenic dissemination of the bacteria.

This case highlights the importance of maintaining clinical vigilance in evaluating patients with complex and evolving symptoms, especially in regions where alternative differential diagnoses are more common. Early recognition and timely imaging are essential to prevent potentially fatal complications.

## Conclusions

Lemierre’s syndrome is a rare but potentially life-threatening complication of oropharyngeal infection that may initially present with nonspecific clinical features, resulting in delays in diagnosis. This case highlights the importance of maintaining a high index of suspicion in patients presenting with worsening sore throat, fever, neck pain, and evolving systemic manifestations, particularly when the clinical progression appears disproportionate to an uncomplicated upper respiratory tract infection.

Prompt recognition with timely radiological evaluation, alongside early initiation of appropriate antimicrobial therapy and selective use of anticoagulation, is critical in improving outcomes and reducing morbidity and mortality, especially in resource-limited settings. This case further emphasizes the importance of clinical vigilance and multidisciplinary management in preventing potentially fatal complications associated with Lemierre’s syndrome.
